# Association of Health Utility Score with Physical Activity Outcomes in Stroke Survivors

**DOI:** 10.3390/ijerph18010251

**Published:** 2020-12-31

**Authors:** Masashi Kanai, Kazuhiro P. Izawa, Hiroki Kubo, Masafumi Nozoe, Kyoshi Mase, Shinichi Shimada

**Affiliations:** 1 Department of Physical Therapy, Faculty of Nursing and Rehabilitation, Konan Women’s University, Kobe 658-0001, Japan; kanaimasa07@gmail.com (M.K.); nozoe@konan-wu.ac.jp (M.N.); tkjwg268@yahoo.co.jp (K.M.); 2 Department of Public Health, Kobe University Graduate School of Health Sciences, Kobe 654-0142, Japan; itamikousei1@yahoo.co.jp; 3 Cardiovascular Stroke Renal Project (CRP), Kobe 654-0142, Japan; 4 Department of Rehabilitation, Itami Kousei Neurosurgical Hospital, Itami 664-0028, Japan; hiro.k16862@gmail.com; 5 Department of Neurosurgery, Itami Kousei Neurosurgical Hospital, Itami 664-0028, Japan

**Keywords:** health utility, physical activity, quality of life, rehabilitation, stroke

## Abstract

Health-related quality of life (HRQoL) after stroke tends to vary across studies or across stages of stroke. It is useful to use the health utility score to compare HRQoL across studies. Physical activity after stroke also tends to vary similarly. The purpose of the present study was to determine associations between the health utility score and physical activity outcomes in stroke survivors. This cross-sectional study recruited stroke survivors who could ambulate outside, free of assistance. We assessed the health utility score with the EuroQoL 5-Dimension 3-Level questionnaire. The physical activity outcomes were the number of steps taken and duration of moderate-to-vigorous physical activity (MVPA) as measured with an accelerometer. Multiple linear regression analyses were used to determine whether the physical activity outcomes were independently associated with the health utility score. Fifty patients (age: 68.0 years; 40 men, 10 women) were included. Multiple linear regression analysis showed the health utility score to be significantly associated with the number of steps taken (β = 0.304, *p* = 0.035) but not with MVPA. This is the first study to examine the association between the health utility score and objectively measured physical activity in stroke survivors. Promoting physical activity especially by increasing the number of steps taken might be a priority goal in improving a patient’s health utility score after stroke.

## 1. Introduction

Stroke has a direct impact on overall health. The interval between the onset of symptoms and arrival at the hospital can greatly influence the effectiveness of treatment and patient prognosis [1,2]. Stroke survivors are additionally affected by long-term physical and psychosocial well-being [3]. Several studies reported that health-related quality of life (HRQoL) is also altered in stroke survivors. Most stroke survivors have a lower HRQoL than healthy subjects [4,5,6], even when adjusting for confounding factors [6]. De Wit et al. indicated that HRQoL of stroke survivors was more than 1/2 standard deviations below that of a healthy population [4]. However, there are strong associations between levels of HRQoL assessed by the EuroQoL 5-Dimension 3-level (EQ-5D-3L) questionnaire at three months and survival within the first year [7]. Thus, assessing HRQoL may contribute to predicting prognosis after stroke.

HRQoL after stroke tends to vary across studies [7,8] or stages of stroke [9]. Thus, it is useful to use the health utility score to compare HRQoL across different studies. The EQ-5D-3L is particularly useful for calculating the health utility score to compare health over time between different populations [10]. The EQ-5D-3L is utilized for the analysis of cost-effectiveness as a means to use the outcome for quality-adjusted life years (QALY) [11].

Physical activity after stroke also tends to vary across studies or stages of stroke because of factors such as stroke severity, physical function, and environmental factors [12]. Physical activity is just as important as HRQoL as a predictor of prognosis in terms of mortality [13] and recurrent stroke [14,15]. Therefore, promoting physical activity may be one of the most important strategies in improving stroke survival and preventing recurrence. There are several reports on the relationship between HRQoL and physical activity or exercise after stroke. Adaptive physical activity improves mobility function and HRQoL in chronic stroke [16]. Hou et al. suggested thar long-term regular mild exercise such as walking could improve HRQoL after stroke [17]. Based on the above reports, exercise intervention might have a positive effect on HRQoL. In a cross-sectional study, Rand et al. reported that HRQoL correlated significantly with the amount of daily physical activity as measured with an accelerometer and self-reported questionnaire [8]. However, their study did not calculate a health utility score based on HRQoL, so the association of the health utility score with physical activity outcomes was not clear. Previously, we tried to clarify these relationships in a preliminary cross-sectional study [18]. As a result, the health utility score correlated significantly with the number of steps taken in community-dwelling ambulatory patients with stroke. Thus, we concluded that the more the patients with stroke walked, the higher their health utility score would be. However, because the study sample was quite small, we could not conduct a multivariate analysis. Additionally, we could not investigate the association between the health utility score and physical activity intensity. If these associations are clarified even when considering confounding factors, we may be able to develop appropriate methods to increase health utility by promoting physical activity. In addition, the results of the present study might also serve as a steppingstone for examining the relationship between QALY and physical activity in future trials.

We thus hypothesized that the health utility score would be associated with the amount of physical activity or intensity of physical activity in stroke survivors. Therefore, the purpose of the present study was to determine associations between the health utility score and physical activity outcomes in stroke survivors.

## 2. Materials and Methods

### 2.1. Study Design and Participant Recruitment

This cross-sectional study was approved by the research ethics committee of Kobe University Graduate School of Health Sciences (approval no. 690, 20 April 2018). Informed consent was obtained from all patients. Participants were selected from June 2017 to November 2018 at Itami Kousei Neurosurgical Hospital by a medical doctor or physical therapist based on the inclusion criteria. The sample size used in the present study was determined based on the total number of patients seen over the selection period.

The inclusion criteria were a previous history of stroke, ability to ambulate outside free of assistance, and consent to measurement of physical activity. Exclusion criteria were those younger than 18 years of age, patients with dementia or aphasia as evaluated by their primary care physician, those with a modified Rankin Scale score [19] > 3 (moderate to severe disability conditions that require assistance with walking and physical demands) due to musculoskeletal disease, and those with severe cardiopulmonary disease or psychiatric disease such as schizophrenia based on evaluation of the patient’s medical records by a physical therapist.

### 2.2. Clinical Characteristics

Patient characteristics, including age, sex, body mass index (BMI), subtypes of stroke (ischemic or hemorrhage stroke), neurological deficit by the National Institutes of Stroke Scale (NIHSS) [20], time from stroke onset, comorbidities (hypertension, diabetes mellitus, dyslipidemia), handgrip strength, and comfortable walking speed [21] were collected from electronic medical records. The NIHSS is one of the measures of stroke severity and assesses 11 items related to cognition, vision, motor and sensory function, speech and language, ataxia, and inattention. It consists of a score of 0–42, with lower scores indicating milder neurological symptoms and higher scores indicating more severe symptoms. Comfortable walking speed was determined from a 10 m walking test as 10 m/time required in seconds [21]. We used a stopwatch to time the patient’s walking time over a 10 m length of a 14 m walkway. Grip strength was measured using a digital grip strength meter. During the measurement, the patient was instructed to sit in a chair without a backrest and to keep the upper limbs off the side of the body [22]. The grip strength was measured twice on each side, and the maximum value was adopted.

### 2.3. Assessment of Health Utility Score

To assess the health utility score, we used the EQ-5D-3L questionnaire [23]. The EuroQoL-5D-3L was introduced by the EuroQoL group and has been used in Japanese populations. The EQ-5D-3L has an index score as the first component, and patients select outcomes from choices of no problems, some problems, and severe problems (scored 1–3) for the following five dimensions: mobility, self-care, usual activities, pain/discomfort, and anxiety/depression. The responses obtained from the EQ-5D-3L were converted by a physical therapist to a health utility score, which was considered the primary outcome in the present study based on a set of Japanese values [23]. The health utility score assesses HRQoL quantitatively as a fraction of ideal health, with a score of 1 representing perfect health, a score of 0 representing death, and a negative score representing health states worse than death [23].

### 2.4. Physical Activity Measurement

The physical activity outcomes were the number of steps taken and duration of moderate-to-vigorous physical activity (MVPA). We used a Fitbit One 3-dimensional accelerometer (Fitbit, Inc., San Francisco, CA, USA) to measure the physical activity values. The Fitbit One has been used in previous studies of stroke patients [18,19,20,21,22,23,24,25]. After patient enrollment, the device was worn on the waist belt of all patients 24/h day for more than one week, except when bathing or changing clothes. We used the first 7 days (1 week) of continuous data to determine physical activity outcomes in the present study. We confirmed the number of steps, exercise energy expenditure, and duration of activity time after downloading the data files to Fitbit online dashboard software [26]. We calculated the average number of steps (/day) and duration of MVPA (min/day). Duration of MVPA was calculated by the sum of MVPA time at greater than three metabolic equivalents.

### 2.5. Statistical Analysis

The results are shown as median (interquartile range) or as ordinal variables and counts (%) for categorical variables. The Shapiro-Wilk test was used to access normality of the values. Nonparametric analyses were used. Multiple linear regression analyses were used to determine whether the physical activity outcomes were independently associated with the health utility score. The health utility score was the dependent variable, and the independent variables were the physical activity outcome and the following relevant confounding variables that correlated with the health utility score by Spearman correlation coefficient (*p* < 0.05): age, sex, BMI, handgrip strength, and walking speed. To account for the effects of multicollinearity, physical activity outcomes were selected as the independent variables in two separate models (Model 1: number of steps, Model 2: MVPA). A *p*-value of < 0.05 was considered to indicate statistical significance. Statistical analyses were performed with IBM SPSS 25.0 statistical software (IBM SPSS Japan, Inc., Tokyo, Japan).

## 3. Results

### 3.1. Participants and Clinical Characteristics

**Figure 1:**
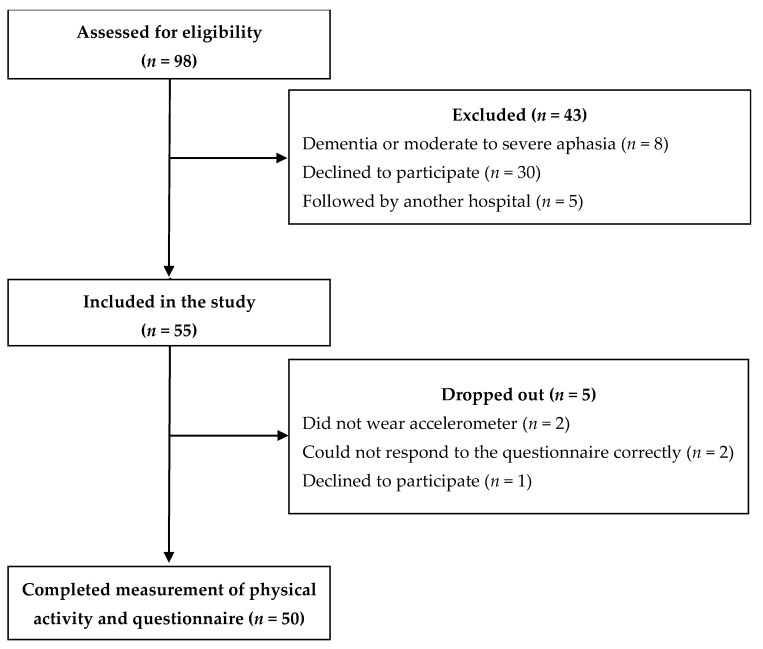
Participant flow in the present study.

Participant flow through the present study is shown in Figure 1. Of the original 98 patients, 55 patients met the inclusion criteria, but five patients later dropped out because they did not wear the accelerometer (*n* = 2), did not respond to the questionnaire correctly (*n* = 2), or declined to participate (*n* = 1). Therefore, the study sample comprised 50 patients. Clinical characteristics of the patients are shown in Table 1.

### 3.2. Health Utility and Physical Activity

The health utility score was 0.77 (0.71–0.85). The physical activity outcomes as indicated by the number of steps taken and the duration of MVPA were 5472.9 (3445.2–7399.9) steps/day and 10.2 (1.7–33.0) min/day, respectively. The health utility score showed a significant positive correlation with BMI, handgrip strength, walking speed, number of steps, and MVPA and a significant negative correlation with sex (female) and NIHSS (Table 2).

The results of the multiple linear regression analyses are shown in Table 3. The health utility score in Model 1 was significantly associated with sex (β = −0.366, *p* = 0.026) and the number of steps taken (β = 0.304, *p* = 0.035). The health utility score in Model 2 was significantly associated only with sex (β = −0.354, *p* = 0.035) and not with MVPA (β = 0.231, *p* = 0.102). In both Model 1 and Model 2, women had lower health utility scores.

## 4. Discussion

### 4.1. Key Findings

To our knowledge, this is the first study to examine the associations between health utility score and objectively measured physical activity in stroke survivors using multivariate analysis. The results indicated that there was an association between the health utility score and the number of steps taken in stroke survivors.

In addition to the EQ-5D-3L, there are other methods for calculating the health utility score from other scales, such as the Short Form 6-Dimension questionnaire [27] and the Health Utility Index 2 (HUI 2) and Health Utility Index 3 (HUI 3) [28]. The HUI 2 overall score includes sensation, mobility, emotion, cognition, self-care, and pain, whereas the HUI 3 includes vision, hearing, speech, ambulation, dexterity, emotion, cognition, and pain. The health utility score was 0.77 in stroke survivors in the present study. Haacke et al. indicated that the health utility scores in long-term survivors of stroke as calculated by the HUI 2, HUI 3, and EQ-5D-3L were 0.67, 0.47, and 0.73, respectively [28]. Post et al. reported that health utility score was 0.64 for patients with minor stroke from a systematic review of the literature [29]. Carod-Artal and Egido also presented several stroke sequels in which the health utility score as assessed by the EQ-5D ranged from 0.60 to 0.70 for minor stroke (NIHSS < 6) [3]. Most of the stroke survivors in the present study suffered strokes of minor severity (median NIHSS score of 1.0), so the health utility score was generally consistent with those of previous studies.

### 4.2. Association of Health Utility Score with Physical Activity Outcomes

Rand et al. reported on the association between HRQoL and physical activity [8], although not the health utility score. We previously reported that the health utility score showed a positive correlation with the number of steps taken in stroke survivors [18]. In terms of the positive correlation between health utility score or HRQoL and physical activity outcomes, the results of the present study support the findings of these two previous studies. The present study added further evidence that there appears to be an association between the health utility score and the number of steps taken, but not with the duration of MVPA after adjusting for confounding factors in stroke survivors.

Macko et al. reported that a structured adaptive physical activity program comprised of mobility, balance, and stretching exercises improves mobility function and QoL in patients with chronic stroke [16]. Another study found that regular mild exercise such as walking or Tai Chi improved QoL after stroke [17]. Grau-Pellicer et al. reported that walking speed was a predictor of community mobility and HRQoL as assessed by the EQ-5D-5L in a population of stroke survivors that included some patients who required supervision to ambulate [30]. Although the health utility score in the present study showed a significant correlation with walking speed, the correlation was not significant after multivariate analysis. Because we only included stroke survivors who could ambulate outside free of assistance, our result might differ from that of the Grau-Pellicer et al. study. Walking activity represented by the number of steps taken is supposed to categorize a community ambulatory level [31], so it is possible that the number of steps may have been altered by engagement with others, and this social interaction might have affected health utility.

Several reports investigated predictors of the health utility score including HRQoL. White et al. suggested that potentially modifiable risk factors such as community participation and stroke-related disability affected HRQoL [9]. Tse et al. reported that the ability to re-engage in work and social activities positively influenced HRQoL in the domains of physical function, participation, and perceived recovery in stroke survivors [32]. Although we could not investigate return to work or return to social activities after stroke in the present patients, the health utility score showed some variability for each stroke survivor, such that the health utility score might have been influenced by work and social activities. Additionally, because these HRQoL or health utility-related factors such as community participation and social activities do not necessarily require high-intensity physical activity, the health utility score was not significantly associated with the duration of MVPA in the present study. Therefore, the health utility score might be more strongly associated with amount of physical activity rather than its intensity in stroke survivors.

The results of this study could be used to suggest that encouragement and support for stroke survivors in order to increase the number of steps for community participation and maintain independence could be a major strategy to increase health utility after stroke. Further study should be conducted to examine QALY and number of steps in stroke survivors.

### 4.3. Limitations

The present study has several limitations. First, we included only stroke survivors who could ambulate outside free of assistance, so generalizability of the results of this study requires caution. The reason we included only minor stroke survivors was because of the accuracy of measuring the number of steps on the instrument. Although health utility after stroke depends on stroke severity [29], physical activity after stroke also depends on stroke severity [33]. Thus, the results of the present study might be partly generalizable, even if the severity and level of mobility of stroke have changed. Second, we could not know the exact wearing time of the accelerometer. The wearing time may have had some impact on the degree of physical activity. Third, because we used the EQ-5D-3L to evaluate the health utility score, there may have been a ceiling effect. In addition, due to the small sample size, it was not possible to compare health utility score by sex difference. Because female stroke survivors tend to have a lower quality of life [34,35], the relationship between the health utility score and physical activity outcome may have needed to be confirmed by sex-specific models. Fourth, we did not evaluate the association of the health utility score with physical activity outcomes considering confounding factors such as depression or anxiety [32,33,34,35,36], which are quite common in stroke survivors and might have been present in the participants of the present study. Finally, in the multivariate regression analysis, we were not able to add a third model that includes number of steps and MVPA due to the small sample size. We need to address these deficiencies in future studies.

## 5. Conclusions

The present study showed that the health utility score was significantly associated with the number of steps taken by stroke survivors but not with the duration of MVPA. The more stroke survivors walked, the higher their health utility score was. Promoting physical activity especially through increasing the number of steps might be a priority goal in improving the health utility score of patients after stroke. Additional study is needed to clarify the association between QALY and number of steps.

## Figures and Tables

**Table 1:** Clinical characteristics.

Characteristic	All Participants (*n* = 50)
Age (years)	68.0 (53.8–77.0)
Sex (male), *n* (%)	40 (80.0)
Body mass index (kg/m^2^)	23.1 (21.8–24.8)
Subtypes, *n* (%)	
Ischemic	32 (64.0)
Hemorrhage	18 (36.0)
NIHSS (score)	1.0 (1.0–2.0)
Time since stroke (months)	4.2 (1.7–6.4)
Comorbidity, *n* (%)	
Hypertension	44 (88.8)
Diabetes mellitus	18 (36.0)
Dyslipidemia	28 (56.0)
Handgrip strength (kgf)	31.9 (24.3–37.6)
Walking speed (m/s)	1.1 (0.9–1.3)

Abbreviation: NIHSS, National Institutes of Health Stroke Scale. Values are shown as median (interquartile range) or as ordinal variables and counts (%) for categorical variables.

**Table 2:** Relation between the health utility score and other characteristics.

	Age	Sex (0, Male; 1, Female)	BMI	NIHSS	Handgrip Strength	Walking Speed	Number of Steps	MVPA
HU score	−0.204	−0.438	0.465	−0.326	0.396	0.429	0.454	0.497
	(0.154)	(0.001)	(0.001)	(0.021)	(0.004)	(0.002)	(0.001)	(<0.001)

Abbreviations: HU, health utility; BMI, body mass index; MVPA, moderate-to-vigorous physical activity; NIHSS, National Institutes of Health Stroke Scale. Values are shown as ρ value (*p* value).

**Table 3:** Multivariate regression analysis for the health utility score.

	Model 1 (Adjusted R^2^ = 0.383)		Model 2 (Adjusted R^2^ = 0.357)	
	β	*p* Value	β	*p* Value
Sex	−0.366	0.026	−0.354	0.035
BMI	0.210	0.109	0.218	0.106
NIHSS	−0.124	0.365	−0.142	0.309
Handgrip strength	−0.158	0.382	−0.082	0.641
Walking speed	0.188	0.182	0.171	0.257
Number of steps	0.304	0.035		
MVPA			0.231	0.102

Abbreviations: BMI, body mass index; MVPA, moderate-to-vigorous physical activity; NIHSS, National Institutes of Health Stroke Scale.
